# Effects of acids used in the microabrasion technique: Microhardness and confocal microscopy analysis

**DOI:** 10.4317/jced.51416

**Published:** 2015-10-01

**Authors:** Núbia-Inocencya-Pavesi Pini, Débora-Alves-Nunes-Leite Lima, Gláucia-Maria-Bovi Ambrosano, Wander-José da Silva, Flávio-Henrique-Baggio Aguiar, José-Roberto Lovadino

**Affiliations:** 1 PhD applicant in Restorative Dentistry, Piracicaba Dental School, University of Campinas – FOP/Unicamp; 2 Professor, Department of Restorative Dentistry, Piracicaba Dental School, University of Campinas – FOP/Unicamp; 3 Professor, Department of Social Dentistry/Statistics, Piracicaba Dental School, State University of Campinas, Piracicaba-SP/Brazil; 4 Professor, Department of Prosthesis, Piracicaba Dental School, State University of Campinas, Piracicaba-SP/Brazil

## Abstract

**Background:**

This study evaluated the effects of the acids used in the microabrasion on enamel.

**Material and Methods:**

Seventy enamel/dentine blocks (25 mm2) of bovine incisors were divided into 7 groups (n=10). Experimental groups were treated by active/passive application of 35% H3PO4 (E1/E2) or 6.6% HCl (E3/E4). Control groups were treated by microabrasion with H3PO4+pumice (C5), HCl+silica (C6), or no treatment (C7). The superficial (SMH) and cross-sectional (CSMH; depths of 10, 25, 50, and 75 µm) microhardness of enamel were analyzed. Morphology was evaluated by confocal laser-scanning microscopy (CLSM). Data were analyzed by analysis of variance (Proc Mixed), Tukey, and Dunnet tests (α=5%).

**Results:**

Active application (E1 and E3) resulted in higher microhardness than passive application (E2 and E4), with no difference between acids. For most groups, the CSMH decreased as the depth increased. All experimental groups and negative controls (C5 and C6) showed significantly reduced CSMH values compared to the control. A significantly higher mean CSMH result was obtained with the active application of H3PO4 (E1) compared to HCl (E3). Passive application did not result in CSMH differences between acids. CLSM revealed the conditioning pattern for each group.

**Conclusions:**

Although the acids displayed an erosive action, use of microabrasive mixture led to less damage to the enamel layers.

** Key words:**Enamel microabrasion, enamel microhardness, confocal laser scanning microscopy.

## Introduction

Enamel microabrasion involves the application of an abrasive coupled with an acid to the affected area. This process allows the outer layer of enamel and, consequently, the stain to be removed through the association between the erosive and abrasive effects (1-4). This technique is used to treat intrinsic stains, such as fluorosis spots (5), inactive white spots due to demineralization (4), and localized hypoplasia (6). The success of the technique depends on the depth of the stained enamel (1,6,7). In general, microabrasion seems to be more efficient when the spots are located on the outermost layers of the dental enamel (6,8).

The microabrasion technique has been modified over the years. Initially, the procedure was performed with 36% hydrochloric acid (9) (HCl), a concentration considered erosive and toxic (4). Heat was applied by a metallic instrument to increase the acid diffusion in the dental structure (9). Croll and Cavanaugh (9) (1986) proposed the mechanical application of a lower concentration of the same acid (18% HCl) in association with pumice as an abrasive. This concentration of acid was able to decalcify the enamel and the stain contained within it. The application of pumice as an abrasive agent with HCl increased the loss of enamel (10). In 1989, it was proposed the use of phosphoric acid (H3PO4) under the same conditions as an efficient technique for enamel microabrasion (11). Currently used microabrasive mixtures include 6% HCl in association with silicon carbide in a commercial presentation and 35% H3PO4 together with pumice as an accessible combination (4), with mechanical application by low-speed rotation (4,5,12,13). These protocols employ lower concentrations of HCl and H3PO4 than have been used in previous protocols, however they use lower pH values, resulting in higher enamel erosion, because of this, the presence of abrasive agent is too important.

In 2008, Paic *et al.* (14) reported that the use of pumice alone was not sufficient enough to remove alone the outer (stained) layer of enamel. This finding showed the importance of the acid in the microabrasion technique. They presumed that the erosive action of the acid was the main factor in enamel removal (10). Although studies have attempted to quantify the enamel alterations that are elicited by microabrasion (1,3,10,14-17), little is known about the effects of the acids that are used in this process on the superficial and deeper layers of enamel. Moreover, in addition to the type, concentration, and pH of the acid, other parameters affect the erosive potential during microabrasion, including the type of abrasive, time of instrumentation, application mode, and force applied (14). Unfortunately, these important factors are poorly described in most studies (3,10,14).

To address these shortcomings in the literature, the purpose of the present study was to evaluate the effects of the acids used, varying their application forms active or passive, in the microabrasion technique on enamel microhardness and morphology.

## Material and Methods

-Preparation of specimens

Seventy bovine incisor teeth without cracks or stains were selected. After the coronary portion had been separated with a double-faced diamond disc (KG Sorensen, Ind. Com. Ltda.; Barueri, SP, Brazil), enamel-dentin blocks of 25 mm2 (5 mm - width x 5 mm - length) were obtained with a precision saw (Isomet 1000; Buehler, Illinois, USA) and a high-concentration diamond disc (4” × 012 × ½, Buehler, Illinois, USA). To obtain flat and standardized enamel surfaces, the blocks were planned by using silicon carbide (SiC) papers of decreasing granulation (#300, #600 e #1200), and the surfaces were polished in a circular polishing machine under water cooling with felts (TOP, RAM, and SUPRA - Arotec, Cotia; SP, Brazil) associated with a diamond paste (6, 3, and 1 µm granulation) and greased with a specific oil (Arotec, Cotia; SP, Brazil). Between the polishing steps and after the final polishing, all slabs were cleaned for 15 min in an ultrasonic bath (Marconi, Piracicaba, SP, Brazil), to remove any rubbish and debries present on the enamel surface. The final thickness of the blocks was standardized about 3 mm, being that 1 mm should be in enamel structure. The specimens were stored in distilled water at 37 °C for 7 days until the beginning of the experiment.

-Enamel surface treatment

The samples were divided into seven groups (n = 10) according to the microabrasive system or the acid used and its form of application:

- Experimental groups:

• Group 1 (E1): Active application of 35% H3PO4 (Ultra EtchTM- Ultradent Products Inc, Utah, USA), performed with a specific rubber cup coupled with a low-rotation electric micromotor;

• Group 2 (E2): Passive application of 35% H3PO4 (Ultra EtchTM- Ultradent Products Inc, Utah, USA);

• Group 3 (E3): Active application of 6.6% HCl (Drogal, Piracicaba, SP, Brazil), performed with specific rubber cup coupled with a low-rotation electric micromotor;

• Group 4 (E4): Passive application of 6.6% HCl (Drogal, Piracicaba, SP, Brazil).

- Control groups:

• Group 5 (C5): Microabrasion with 35% H3PO4 (Ultra EtchTM – Ultradent Products Inc, Utah, USA) associated with pumice (SS White Ltda; Rio de Janeiro, RJ, Brazil), performed with specific rubber cup coupled with a low-rotation electric micromotor;

• Group 6 (C6): Microabrasion with 6.6% HCl associated with sílica (OpalustreTM – Ultradent Products Inc, Utah, USA), performed with specific rubber cup coupled with a low-rotation electric micromotor;

• Group 7 (C7): No treatment.

For the microabrasive system composed of H3PO4 and pumice (C5), equal parts of each component were measured with a dosage spoon (0.240 g) and mixed. All of the components were placed on the enamel surface with a syringe until the sample was covered; the amount required corresponded to 0.0200 g for the groups treated with acid and abrasive and 0.0150 g for the groups treated with acid only.

The active application and the microabrasion was performed with specific rubber cups (OpalcupsTM – Ultradent Products Inc, Utah, USA) coupled with a low-rotation electric micromotor (LB-2000, Beltec Indústria e Comércio de Equipamentos Odontológicos Ltda, São Paulo, SP, Brazil); with standardized rotation estimated about 13000 rpm. The passive application consisted in just applied the acid on the enamel surface. All of the treatments were performed with 10 applications of 10 s each. After each application, the enamel surface was rinsed and dried for 10 s with a dental sprayer and compressed air, respectively.

-Microhardness test

The Knoop surface microhardness (SMH) and cross-sectional microhardness (CSMH), at a load of 25 g with an indentation time of 10 s, of the enamel were determined with a microhardness tester (Shimadzu HMV-2000, Shimadzu Corp., Kyoto, Japan). The SMH was tested twice, before (initial) and after (final) the microabrasion procedure. For the SMH analysis, five indentations spaced 100 µm apart were made in the center of the enamel block. For the CSMH tests, the blocks were longitudinally sectioned through the center. One of the halves had its cut face exposed and gradually polished with SiC papers, felts, and diamond paste, as previously explained. Four rows of three indentations spaced 100 µm apart were made at 10, 25, 50, and 75 µm from the outer enamel surface. The length of each indentation was measured with an optical analysis system coupled with the microhardness tester. The mean values at all three measuring points at each distance from the surface were averaged.

-Confocal laser-scanning microscopy (CLSM)

Slices were obtained from the other half of the cut samples that were not used for the CSMH analysis. These slices were polished with SiC papers of decreasing granulation, surface-polished with felts (TOP, RAM, and SUPRA - Arotec, Cotia; SP, Brazil) associated with a diamond paste of decreasing granulation (6, 3, and 1 µm, respectively), and greased with a specific oil (Arotec, Co-tia; SP, Brazil). This process resulted in 0.3-mm-thick slices. The specimens were washed for 15 min in an ultrasonic tub (Marconi, Piracicaba, São Paulo, Brazil) between each of these procedures and after the final polishing step.

For CLSM analysis, the specimens were stored in freshly prepared 0.1 mMrhodamine B (Aldrich Chem. Co., Milwaukee, Wisc., USA) for 1 h, without further rinsing (18-20). They were examined with a Leica TCS SP2-SE microscope (Leica Microsystems Gmbh, Manhein, Germany) in fluorescent mode. A HeNe 543 gas laser was used as the light source. The intensity of the excitation light and the amplification of the photomultiplier were kept constant during the investigation period. The detected light was conducted through a 543-mm long-pass filter; thus, the emitted fluorescent light was discriminated from the reflected and the scattered light. The visualized layer was selected 10 μm below the sample surface, and images were recorded with an oil immersion objective (40X, numerical aperture 1.25).

-Statistical analysis

The data obtained were submitted to statistical analysis. After exploratory data analysis, variance analysis for repeated measures using PROC MIXED was applied, followed by the Tukey-Kramer and Dunnett tests. The significance level was 5%.

## Results

-SMH analysis

The results of the SMH analysis are shown in  Table 1. In relation to the control groups, the treatments with H3PO4 and pumice (C5) or HCl and silica (C6) did not resulted in significantly reduced enamel SMH means compared to the initial measurement and the group without treatment (C7) (*p*>0.05). For the groups treated by active application of H3PO4 (E1) and HCl (E3), the SMH values were statistically different from those of all other groups (*p*<0.05); however, no differences in SMH between E1 and E3 were observed. Groups with the passive application of H3PO4 (E2) and HCl (E4) showed the lowest SMH results. The SMH values of E2 and E4 were significantly different compared to the experimental groups with active application and the control groups; however, no significant differences in SMH between E2 and E4 were observed.

**Table 1:**
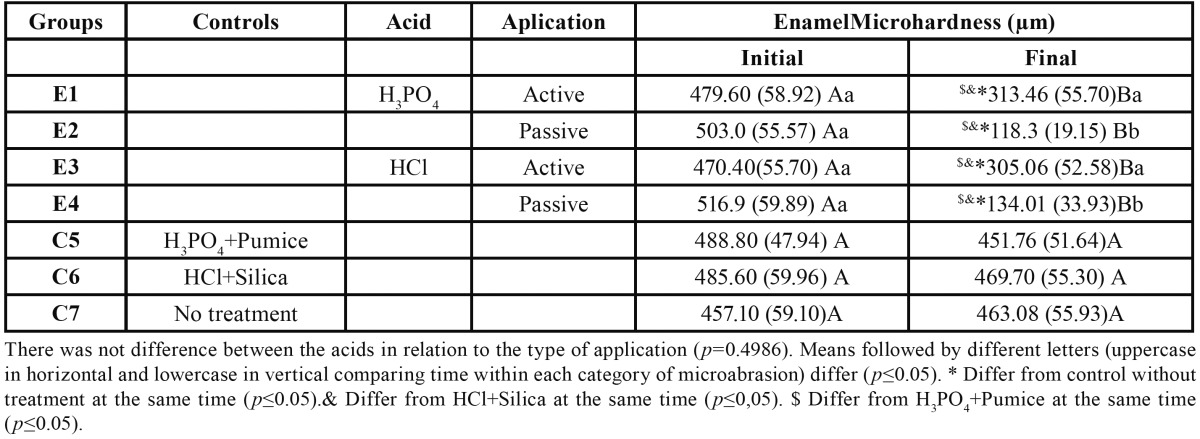
Results for Knoop surface microhardness (SMH) according to the treatment group (mean ± SD, n = 10)

-CSMH analysis

**Figure 1:**
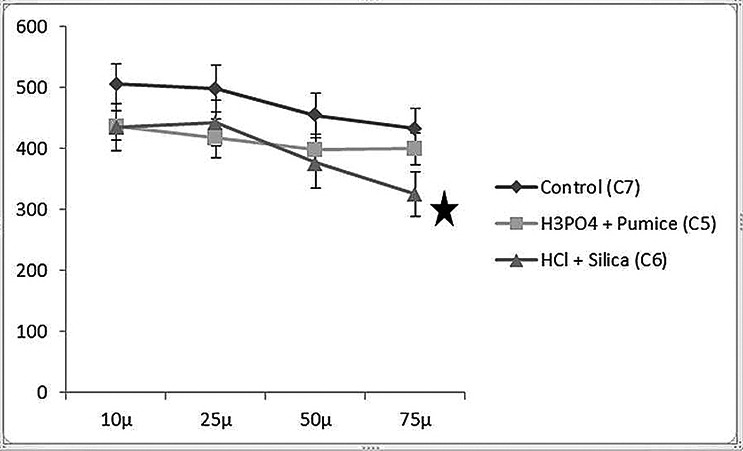
Mean Knoopcross-sectional microhardness (CSMH) values according to the treatment and distance (µ) from the surface for the control groups (bars denote SD, n = 10). Star, significantly different from H3PO4+pumice).

**Figure 2:**
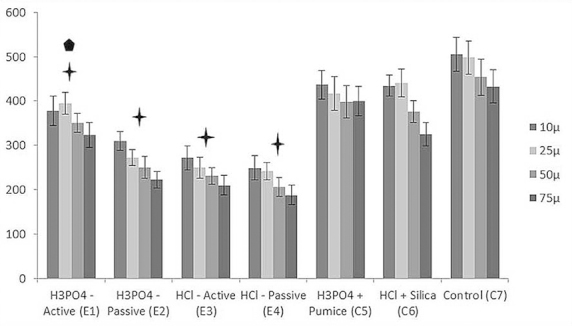
Mean Knoopcross-sectional microhardness (CSMH) values according to the treatment and distance (µ) from the surface (bars denote SD, n = 10). Plus sign, significantly different compared to controls; Pentagon, significantly different compared to actively applied HCl.

Figures 1 and 2 show the results of the CSMH analysis. The groups treated by microabrasive systems with acid, abrasive, and rotation (C5 and C6) showed reduced CSMH values in all of the deeper layers of the enamel (10, 25, 50, and 75 µm), with statistical differences compared to the group with no treatment (C7) (*p*<0.05). The group treated with HCl and silica (C6) showed significantly lower CSMH values at 75 µm than the group treated with H3PO4 and pumice (C5). In C5, no differences in CSMH between the analyzed depths were observed, whereas C6 showed differences in CSMH between the deeper layers (50 and 75 µm) and the superficial layers.

For the groups with active application of H3PO4 (E1) and HCl (E3), the mean CSMH was reduced in all of the enamel layers, with statistical differences compared to the control groups (C5, C6, and C7). In all of the layers analyzed, the reduction of CSMH was significantly higher with active application of H3PO4 compared to that of HCl. The groups with passive application presented significant differences in CSMH compared to the controls, but no significant differences in CSMH between the acids were observed.

For both acids and application types (active/passive), there were significant differences in CSMH between the superficial and deeper layers. All of the mean CSMH values of experimental groups treated with HCL (E3 and E4) differed from those of the group treated with HCl and silica (C6). Similarly, the mean CSMH values of experimental groups treated with H3PO4 (E1 and E2) differed from that of the control treated with H3PO4 and pumice (C5). The experimental groups showed greater reduction of the CSMH as compared to the corresponding positive control group. All of experimental group treatments resulted in differences in CSMH compared to the control group without treatment (C7).

-Morphology analysis by CLSM

**Figure 3:**
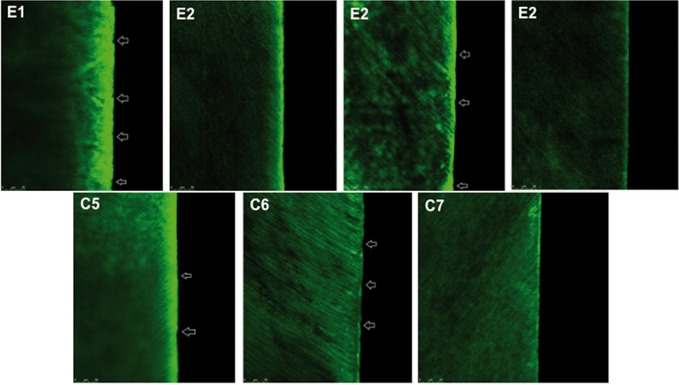
Representative images of the groups byconfocal laser-scanning microscopy (CLSM). E1 and E2: Groups treated with 35% H3PO4 and pumice with active and passive application, respectively; E3 and E4: Groups treated with 6.6% HCl with active and passive application, respectively; C5 and C6: Groups treated with microabrasion using 35% H3PO4 and pumice and HCl and silica, respectively. Images show points of localized surface microwear (active application groups) and transversal exposure of the enamel prisms (passive application groups).

Figure 3 shows the results of the CLSM analysis. The subsurface structures remained unchanged after all of the treatments. All of the groups treated with acids or with microabrasive systems showed different surface conditioning patterns compared to the group without treatment (C7). In the case of the groups treated with microabrasive systems (H3PO4 + Pumice – C5 and HCl + Silica – C6), localized surface microwear was observed. Surface microwear was also seen in the groups with active application of H3PO4 (E1) and HCl (E3). CLSM images for the groups with passive application of H3PO4 (E2) and HCl (E4) showed transversal exposure of the enamel prisms, which was a result of the acid conditioning. The light-colored seam observed at the top of the specimens in almost all of the images demonstrated the light-reflecting properties of the uppermost surface regions of the enamel slabs. This effect might be due to a smear layer induced by surface polishing.

## Discussion

No statistically significant difference was observed between the SMH results before and after enamel microabrasion with 35% H3PO4 and pumice (C5) or 6% HCl and silica (C6 –HCl and silica - Ultradent Products), as previously described (15,17). Studies have suggested the existence of an “abrosion effect”, whereby the erosive action of the acid couples with the abrasive action to compact the mineralized tissue within the organic area (1). Through this process, the outer layer of prism-rich enamel is replaced with a densely compacted, prism-free region (4,5), creating a fluorapatiterich surface layer (8). The Knoop CSMH means were significantly lower in all enamel layers in the positive control groups treated with microabrasion (C5 and C6) compared to the group with no treatment (C7). This finding demonstrates the erosive power of the acids. The only significant difference in CSMH means between the positive control groups was observed between the 75 µm layer from C5 and C6. This result reflects the higher erosive power of HCl compared to H3PO4 and its action in deep layers, which could be related to the lower pH value of HCl (-0.14) compared to H3PO4 (0.79) (19).

The experimental groups with active application of H3PO4 (E1) and HCl (E3) showed significantly reduced SMH results compared to the control groups with (C5 and C6) and without (C7) treatment. This finding shows the importance of the presence of the abrasive agent for neutralizing the erosive action of the acids. At the same time, the combined use of abrasive and erosive actions is key (1,4,5,15-17), as studies have shown that the use of pumice alone is insufficient to remove the outer enamel layer (14). The mean CSMH results progressively decreased with increasing depth, with differences between the acids. This finding was probably due to enamel topography, because enamel layers nearer to the dentin-enamel junction are more susceptible to demineralization than other layers (21).

For the experimental groups with active application, the CSMH results were lower for HCl (E3) than those for H3PO4 (E1), probably due to the lower pH of HCl (17). Thus, the low pH values of both acids coupled with mechanical application (and, consequently, pressure) led to considerable enamel erosion (19,22). The groups treated by passive application of the acids (E2 and E4) presented the lowest values of SMH ( Table 1) and CSMH (Fig. 2). In all of the enamel layers, the microhardness values in E2 and E4 were significantly different from those of the other experimental and control groups. This finding suggests that the use of mechanical application helps to guarantee the scattering and renovation of the acid on the enamel, without allowing the erosive substance to remain on the tooth surface for too long.

No significant differences were observed between the acids after passive application, consistent with previous findings that the use of 35% H3PO4 or 6.6% HCl does not result in differences with respect to mineral loss (19,23). With passive application, the lowest mean microhardness results were observed in the outermost layer, indicating that the effect of the acid on the surface microhardness is stronger than that in the deeper layers. Similarly, Honório *et al.* (24) (2010) stated that erosive demineralization is restricted to the surface until enamel loss from this surface occurs. Therefore, acids can reach the deepest layers of the enamel only after they cause changes in the surface. In the absence of mechanical application, the viscosity of the acidic gels retards their penetration into the enamel and, therefore, the erosion effect (19).

The effect of the acids on enamel morphology was analyzed by CLSM. This microscopy technique can reveal the enamel ultrastructure through the light reflection and transmission properties of the dental structure. CLSM provides a tremendous complementary technique to surface observation (19,25), but it has limited sensitivity to detect lesions of less than approximately 15 µm in depth (23,24). Based on previous findings (19,20,26), rhodamine B fluorescent dye was used in this study because it can penetrate into the enamel voids created during demineralization (20).

The CLSM images showed that the subsurface enamel was unchanged after treatment with microabrasive systems or acids, but the experimental groups displayed differences in the structural changes. The CLSM images for groups with mechanical application showed evidence of potential microwears, which were possibly the result of the use of an abrasive agent (C5 and C6) or mechanical application (E1, E3, C5, and C6) during the treatment. Previous studies have also shown that increased pressure (12) and the presence of abrasive (10,14) result in increased enamel loss. The images from the groups with passive application of the acids (E2 and E4) showed the transversal exposure of the enamel prism, which was possibly due to the diffusion of the acid through the enamel.

The clinical success of the microabrasion technique has been well-documented (1,4-6,13). As related in previous studies (10,12,14) and in the present study, when planning the microabrasion procedure, the clinician should consider the effects of the technique on the enamel. Although there were no significant differences between the microabrasive systems used with respect to the enamel microhardness, when the acids were applied alone, both H3PO4 and HCl showed an erosive action that was able to reduce the mineral content of the tooth structure.

Overall, the results of this study showed that the neutralization action of the abrasive was useful for preventing further damage to the dental enamel. Additional investigations of the proportion between the acid and abrasive agent in microabrasive systems should be performed. Such studies should focus on the development of an ideal system that allows the effective removal of the changed superficial enamel, while safety preserving the enamel characteristics in the deeper layers.

The results of this study verify the erosive power of H3PO4 and, especially, HCl. Compared to H3PO4, the active application of HCl resulted in significantly greater reductions in the microhardness results in the deeper enamel layers. The use of microabrasive systems containing acid and abrasive compounds combined with mechanical application can allow the efficient reduction of enamel damage in the superficial and deeper layers, thereby maintaining the enamel integrity as such as possible.
